# Self-Harm Before and Six Months After Obesity Surgery

**DOI:** 10.1007/s11695-024-07439-3

**Published:** 2024-08-13

**Authors:** Tobias A. Thomas, Katja Tilk, Katharina Klassen, Melanie Pommnitz, Ruth Wunder, Julian W. Mall, Hinrich Köhler, Martina de Zwaan, Günther Meyer, Thomas P. Hüttl, Astrid Müller

**Affiliations:** 1 Department of Psychosomatic Medicine and Psychotherapy, Hannover Medical School, Carl-Neuberg-Straße 1, 30625 Hanover, Germany; 2 Department of General, Visceral, and Bariatric Surgery, DRK Krankenhaus Clementinenhaus, Lützerodestr. 1, 30161 Hanover, Germany; 3 Department of General, Visceral, and Bariatric Surgery, KRH Nordstadt, Haltenhoffstr. 41, 30167 Hanover, Germany; 4 Department of General, Visceral, and Bariatric Surgery, Herzogin Elisabeth Hospital, Leipziger Straße 24, 38124 Brunswick, Germany; 5 Department of General, Visceral, and Bariatric Surgery, AMC-WolfartKlinik, Waldstraße 7, 82166 Gräfelfing, Germany; 6 Department of General, Visceral, and Bariatric Surgery, Dr. Lubos Kliniken Bogenhausen, Denninger Str. 44, 81679 Munich, Germany

**Keywords:** Self-harm, Eating pathology, Anxiety, Depression, Alcohol use, Obesity surgery

## Abstract

**Purpose:**

Previous research on obesity surgery (OS) showed that patients do not only experience weight loss but also improvements in certain mental health outcomes (e.g., depression) after OS. However, self-harm behaviors might increase after OS. Regarding self-harm, the literature is mostly limited to studies using data from hospital or emergency room charts. This longitudinal study examined self-reported self-harm behaviors and potential psychopathological correlates before and after OS.

**Materials and Methods:**

Pre-surgery patients (*N* = 220) filled out a set of questionnaires before and approximately six months after OS. Self-harm behaviors were captured with the Self-Harm Inventory. The assessments further included standardized instruments to measure symptoms of depression, anxiety, eating disorders, alcohol use, and suicidal ideations.

**Results:**

Any self-harm was reported by 24.6% before and by 25.0% after OS. No differences in the number of self-harm behaviors or prevalence of any self-harm before and after OS were found. Overall, 11.4% experienced self-harm behaviors at both times. A subset showed self-harm behaviors only before (13.2%) OS and another subset only after OS (13.6%). These two groups were about the same size. Self-harm behaviors showed strong associations with psychopathology after OS, especially with depression and suicidal ideation.

**Conclusion:**

No increase in self-harm behaviors after OS emerged. Still, a subgroup showed self-harm behaviors after OS closely linked to further psychopathology. This mirrors the need to implement screening for self-harm before *and* after OS into OS care. Further studies with longer follow up periods are needed to extend these findings.

**Graphical Abstract:**

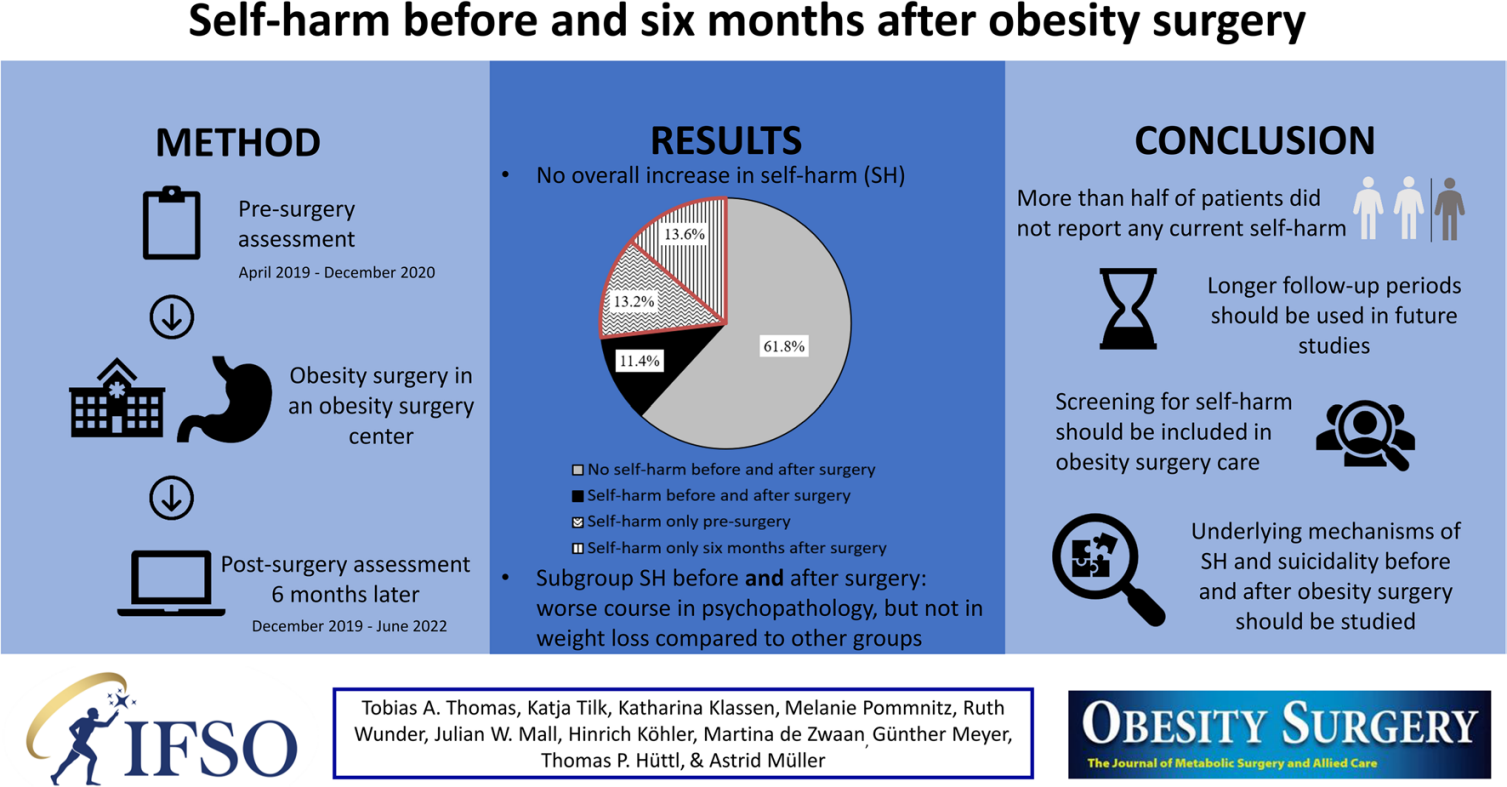

## Introduction

Obesity is a disease with an overall high prevalence (e.g., [1]) and is closely associated with psychopathology such as depression (*depreobesity*, e.g., [2]), anxiety disorders (e.g., [3]) and eating disorders (e.g., [4]). In the vast majority of cases, obesity surgery results in positive outcomes, including sustained weight loss, metabolic benefits, improved quality of life and reduced mortality [5]. However, longitudinal studies suggest that the risk of suicide and self-harm (including risky alcohol use) increases after obesity surgery [6–8]. Likewise, observational cohort studies indicate a higher incidence of self-harm emergencies [9, 10] and suicide attempts in individuals who underwent bariatric surgery than in nonsurgical samples [6, 11]. Most of these studies used data from hospital or emergency room charts for self-harm and/or suicidal attempts defined by ICD-10 codes (e.g., X60-X84, Y10-34, Y870) [9, 10, 12–15]. It is questionable whether the results studies based on patient charts reflect the actual incidence of self-harm behaviors given that not all individuals who harmed themselves contact medical services afterwards or present in emergency settings [16, 17]. Gordon et al. [18] used a different approach and examined self-harm/suicidal ideation prospectively before and after surgery in a large cohort (*N* = 2,217) by using the following item of the Beck Depression Inventory: “I don’t have any thoughts of harming myself” [19]. The differences in BDI-based prevalence rates of self-harm/suicidal ideation between pre-surgery (5.3%; 95% CI [3.7, 6.8]) and 12 months post-surgery (3.8%; 95% CI [2.5, 5.1]) as well as between pre-surgery and 5 years after surgery (6.6%; 95% CI [4.6, 8.6]) were not significant [18]. However, it remains unclear to what extent the findings indicate real self-harm or suicidal attempts.

The Self-Harm Inventory (SHI) developed by Sansone et al. [20] is a self-report instrument to assess a broad range of 22 self-harm behaviors. Past studies used the questionnaire to explore the lifetime prevalence of self-harm in obesity surgery candidates [21, 22]. Sansone et al. [22] examined a sample of 121 pre-surgery patients from the US and found that almost half of the sample (46%) admitted any self-harm during the lifespan. Likewise, about every second patient (52%) in a German pre-surgery sample (*N* = 139) was affected by any lifetime self-harm behavior, which was less frequent than in a community control group with obesity class 2 or 3 (*N* = 122, lifetime prevalence 64%) [21].

The current longitudinal study builds on the aforementioned cross-sectional investigations [21, 22] by using the SHI [20] in obesity surgery patients before and six months after surgery. The main aim was to shed more light on possible short-term changes in self-harm. Another aim was to explore how self-harm is related to mental health disorders that occur frequently in individuals seeking obesity surgery and may improve relatively quickly after surgery, particularly depression, anxiety and eating disorders (including addiction-like eating) [7, 23–25]. Follow-up studies found rapid improvement in depressive [23, 25–27] and eating (including addiction-like eating) [23, 28, 29] disorder symptoms 6 through 24 months after obesity surgery. Results regarding short-term post-surgery changes in symptoms of anxiety disorder [26, 30–32] are less conclusive with most studies indicating neither improvements nor deterioration up to one year following surgery. In addition, the current study addressed alcohol use given that some individuals are at-risk of developing problems with alcohol following bariatric surgery [33]. Moreover, gender effects were examined, as women appear to be more likely to suffer from self-harm than men (especially cutting and suicide attempts) [34–36]. Due to the exploratory nature of the current study and the lack of literature on short-term postoperative changes in self-harm and the association between self-harm and psychopathology before and relatively soon after obesity surgery, no formal hypotheses were drawn.

## Methods

### Procedure

The current study was carried out in the context of a longitudinal obesity surgery study concerning weight-related quality of life, post-surgery dumping syndrome and self-harm (preregistered at German Clinical Trials Register, DRKS00016677). The initial sample included 419 adults seeking obesity surgery (t0) [37]. Of these, 220 individuals took part in the post-surgery follow-up (t1). Findings addressing the relationship between post-surgery dumping and psychosocial variables in this sample were published elsewhere [38].

Recruitment of participants took place during the pre-surgery psychosocial evaluation or medical examination (t0) at three German hospitals (Hannover Medical School, *n* = 92; Dr. Lubos Kliniken Bogenhausen, *n* = 92; AMC-WolfartKlinik Graefeling, *n* = 36) (between April 2019 and December 2020. Inclusion criteria were age ≥ 18 years, obesity class II or III and sufficient command of the German language. Exclusion criteria were presence of mental retardation or psychosis and acute suicidality. Assessments were completely independent from pre-surgery psychosocial evaluations and pre- and post-surgery medical care. For the 6-months follow-up (t1), patients were invited via email to participate. The follow-up assessments were conducted between December 2019 and June 2022 (months after surgery current sample, *M* = 5.99, *SD* = 1.39, *Mdn* = 6.00).

The study protocol met ethical and legal aspects of research involving human subjects in accordance with the Declaration of Helsinki (Institutional Review Board Approval No. 8133_BO_S_2018, Hannover Medical School, Germany). All participants gave written informed consent.

### Participants

The present study sample consisted of 220 participants, mean age 40.99 years (*SD* = 11.07), 76% female, mean BMI at t0 49.48 kg/m^2^ (*SD* = 6.32) and at t1 36.52 kg/m^2^ (*SD* = 5.56; *t*(219) = 53.48, *p* < 0.001, *d* = 6.07). In terms of age, gender and BMI, participants (t0, *n* = 220) were similar to those in the initial pre-surgery sample (*N* = 419; age, *M* = 40.97 years, *SD* = 11.09; 74% female; BMI, 49.37 kg/m^2^, *SD* = 7.04) [37].

Obesity surgeries were conducted in five different obesity centers (Dr. Lubos Kliniken Bogenhausen, Munich, *n* = 92; KRH-Klinikum Nordstadt, Hanover, *n* = 59; AMC-WolfartKlinik Graefeling, *n* = 36; DRK-Krankenhaus Clementinenhaus, Hanover, *n* = 25; Herzogin Elisabeth Hospital, Brunswick, *n* = 8). Sleeve gastrectomy was the most common procedure (*n* = 152), followed by bypass surgery (*n* = 68).

### Instruments

Self-harm was measured with the German translation (kindly provided by Dr. Paul Plener, Ulm University) of the SHI [20], which comprises 22 dichotomous items (yes = 1, no = 0). Given the one-factor structure of the German SHI [34], a total score was computed by adding up all items answered with ‘yes’ (range 0–22; present sample Cronbach’s α_t0_ = 0.72, α_t1_ = 0.77). SHI total scores ≥ 1 were indicative for reporting any self-harm. For the current study, we adjusted the SHI reference period to the past four weeks prior to assessment (‘Have you in the last four weeks …?’ instead of ‘Have you ever …?’) in order to capture the point prevalence (instead of lifetime prevalence like in the original SHI) and to align the four-week-period with the other questionnaires.

The German version [39] of the Hospital Anxiety and Depression Scale (HADS; [40]) was used to determine symptoms of anxiety and depressive disorders. The HADS consists of two subscales representing anxiety (HADS_Anx_, 7 items, Cronbach’s α_t0_ = 0.69, α_t1_ = 0.82 in current study) and depression (HADS_Depr_, 7 items, α_t0_ = 0.72, α_t1_ = 0.85 in current study).

Suicidal ideations were assessed with item 9 of the Patient Health Questionnaire depression module (PHQ-9) [41, 42], which indicated good ability to screen for suicidal ideations in previous studies [43, 44]. The time window for the response was adjusted slightly from two to four weeks (‘How often have you been bothered during the past four weeks by thoughts that you would be better off dead or of hurting yourself in some way?’; 0 = not at all, 1 = several days, 2 = more than half the days, 3 = nearly every day). To explore changes from pre-surgery to post-surgery, data were dichotomized by collapsing the first two categories into ‘no or rare suicidal ideations’ (= 0) and categories 2 and 3 into ‘frequent suicidal ideations’ (= 1).

Eating disorder pathology was assessed with the global score of the German version [45] of the Eating Disorder Examination Questionnaire (EDE-Q) [46] which includes 22 items answered on a 0 to 6 Likert scale and referring to the past 4 weeks (α_t0_ = 0.81, α_t1_ = 0.91 in current study).

Addiction-like eating was captured with the German version [47] of the Yale Food Addiction Scale 2.0 (YFAS 2.0) [48]. The YFAS 2.0 consists of 35 items (from 0 = never to 7 = always). A symptom score was computed which reflects the number of fulfilled criteria indicative for the addictive consumption of highly palatable foods within the past four weeks (range, 0–11; α_t0_ = 0.90, α_t1_ = 0.85 in current study).

Alcohol use was measured with the German version [49] of the 10-item Alcohol Use Disorder Identification Test (AUDIT) [50]. The AUDIT total score ranges from 0 to 40 (α_t0_ = 0.83, α_t1_ = 0.82 in current study).

For all questionnaires, higher scores indicate more psychopathology.

### Statistical Analysis

Statistical analyses were conducted with SPSS® version 28.0 (IBM Corp., Armonk, NY, USA). The SHI, YFAS 2.0 and PHQ-9 suicidality item data were not normally distributed. Therefore, all within-group changes from before (t0) to six months after (t1) surgery were explored using the non-parametric Wilcoxon test for continuous variables and the McNemar’s test for categorical variables. For continuous variables, effects sizes *r* were calculated [51]. A value < 0.25 was considered a small effect, between 0.30 and 0.40 a medium effect, and values > 0.40 a strong effect [52]. Groups that reported self-harm at t0 and t1, only at t0, only at t1 and at neither t0 nor t1 were compared at baseline and follow-up using Kruskal–Wallis tests. Pairwise comparisons were also provided with an adjusted *p*-value to correct for multiple testing. The relationships of self-harm with BMI and other psychopathological variables (*n* = 6) were analyzed using two-tailed Spearman correlations with Bonferroni-correction (α_adj_ = 0.05/6) [53]. Correlations of 0.10, 0.20, and 0.30 were considered as small, moderate, and large [54]. Fisher’s *r* to *z* transformation was used to examine whether correlation coefficients between two independent groups (e.g., men and women) were statistically significant.

## Results

### Self-Harm and Other Psychopathological Behaviors Prior and 6 Months After Obesity Surgery

Changes from before to after surgery in SHI means and other continuous variables for the total sample are shown in Table 1. The number of self-harm behaviors did not differ between t0 and t1. All other variables improved significantly, with large effects for eating pathology, anxiety and depressive symptoms, and a small effect for alcohol use. Frequent suicidal ideations (dichotomized item 9 PHQ depression module) were reported by 36 participants at t0 and 6 participants at t1 (16.4% vs. 2.7%, respectively, exact McNemar's test, *p* < 0.001).

**Table 1:** Changes in self-harm and other psychopathological variables from pre-surgery (t0) to six months after surgery (t1)

		t0	t1	Within-group comparison		
	*n*	*Mdn (Min/Max)*	*Mdn (Min/Max)*	*z*	*p*	*│r*│
SHI	220	0.00 (0.00/8.00)	0.00 (0.00/11.00)	0.25	.804	0.02
EDE-Q	220	3.04 (0.54/5.19)	1.90 (0.15/5.57)	− 10.27	< .001	0.69
YFAS 2.0	220	4.00 (0.00/11.00)	0.00 (0.00/10.00)	− 11.04	< .001	0.74
AUDIT	220	1.00 (0.00/24.00)	0.00 (0.00/27.00)	− 3.78	< .001	0.25
HADS-A	220	8.00 (0.00/17.00)	4.00 (0.00/19.00)	− 9.95	< .001	0.67
HADS-D	220	9.00 (0.00/19.00)	2.00 (0.00/16.00)	− 11.89	< .001	0.80

*SHI* Self-Harm Inventory; *EDE-Q* Eating Disorder Examination Questionnaire mean; *YFAS* Yale Food Addiction Scale; *AUDIT* Alcohol Use Disorder Identification Test; *HADS-A* Hospital Anxiety and Depression Scale, subscale anxiety; *HADS-D* Hospital Anxiety and Depression Scale, subscale depression

For all variables, separate analyses for men and women indicated similar effects to the total sample.

Table 2 details admitted self-harm behaviors before and after surgery. Item 20 ‘tortured yourself with self-defeating thoughts’ was most often answered in an affirmative manner (‘yes’ at t0, 20.5%; t1, 18.2%). The remaining self-harm behaviors were either not reported at all or very rarely. This also applies to item 18 ‘attempted suicide’, which was mentioned by only one woman before surgery. In descriptive terms, there were no gender differences. Due to the small frequencies of self-harm behaviors, we refrained from making a statistical comparison between men and women, except for item 20, which was answered with ‘yes’ relatively often. Frequencies of ‘yes’ responses for this item did not differ between men and women (t0, X^2^(1) < 0.01, *p* = 0.950; t1, X^2^(1) = 0.07, *p* = 0.795).

**Table 2:** Self-harm behaviors pre-surgery (t0) and six months after surgery (t1) measured with the Self-Harm Inventory (16) in the total sample (*N* = 220), and separately for men (*n* = 53) and women (*n* = 167)

	t0, *n* (%)			t1, *n* (%)		
SHI	Total	Men	Women	Total	Men	Women
1. Overdosed	1 (0.5)	-	1 (0.6)	1 (0.5)	-	1 (0.6)
2. Cut yourself on purpose	3 (1.4)	1 (1.9)	2 (1.2)	2 (0.9)	1 (1.9)	1 (0.6)
3. Burned yourself on purpose	1 (0.5)	-	1 (0.6)	1 (0.5)	-	1 (0.6)
4. Hit yourself	1 (0.5)	1 (1.9)	-	1 (0.5)	-	1 (0.6)
5. Banged your head on purpose	-	-	-	-	-	-
6. Abused alcohol	8 (3.6)	3 (5.7)	5 (3.0)	9 (4.1)	4 (7.5)	5 (3.0)
7. Driven recklessly on purpose	5 (2.3)	4 (7.5)	1 (0.6)	8 (3.6)	3 (5.7)	5 (3.0)
8. Scratched yourself on purpose	9 (4.1)	-	9 (5.4)	4 (1.8)	-	4 (2.4)
9. Prevented wounds from healing	7 (3.2)	1 (1.9)	6 (3.6)	9 (4.1)	2 (3.8)	7 (4.2)
10. Made medical situations worse on purpose	5 (2.3)	2 (3.8)	3 (1.8)	6 (2.7)	1 (1.9)	5 (3.0)
11. Been promiscuous	3 (1.4)	1 (1.9)	2 (1.2)	3 (1.4)	-	3 (1.8)
12. Set yourself up in a relationship to be rejected	-	-	-	1 (0.5)	-	1 (0.6)
13. Abused prescription medication	1 (0.5)	1 (1.9)	-	2 (0.9)	1 (1.9)	1 (0.6)
14. Distanced yourself from God as punishment	2 (0.9)	2 (3.8)	-	3 (1.4)	1 (1.9)	2 (1.2)
15. Engaged in emotionally abusive relationship	3 (1.4)	1 (1.9)	2 (1.2)	3 (1.4)	1 (1.9)	2 (1.2)
16. Engaged in a sexually abusive relationship	1 (0.5)	-	1 (0.6)	1 (0.5)	-	1 (0.6)
17. Lost a job on purpose	-	-	-	3 (1.4)	1 (1.9)	2 (1.2)
18. Attempted suicide	1 (0.5)	-	1 (0.6)	-	-	-
19. Exercised an injury on purpose	3 (1.4)	1 (1.9)	2 (1.2)	1 (0.5)	-	1 (0.6)
20.Tortured yourself with self-defeating thoughts	45 (20.5)	11 (20.8)	34 (20.4)	40 (18.2)	9 (17.0)	31 (18.6)
21. Starved yourself to hurt yourself	-	-	-	-	-	-
22. Abused laxatives to hurt yourself	-	-	-	2 (0.9)	-	2 (1.2)

The prevalence of any self-harm (i.e. SHI_total_ ≥ 1) did not differ significantly between t0 and t1, with 24.6% (95% CI [18.8, 30.3]) of the total sample reporting at least one self-harm behavior pre-surgery, and 25.0% (95% CI [19.2, 30.8]) post-surgery. No differences were found between men and women at t0 (men, 28.3%, 95% CI [15.8, 40.8]; women, 23.4%, 95% CI [16.9, 29.8]) and t1 (men, 26.4%, 95% CI [14.2, 38.7]; women, 24.6%, CI [18.0, 31.2]).

**Fig. 1:**
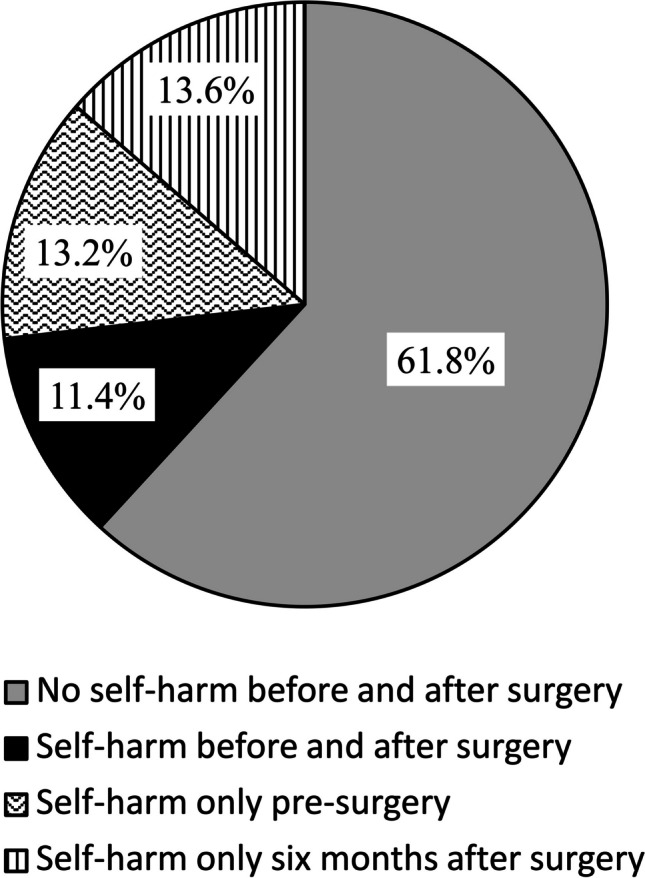
Any self-harm six months after surgery (t1) relative to pre-surgery (t0), *N* = 220

Figure 1 combines the data on any self-harm at t0 and t1. Most participants did not report any self-harm, neither at t0 nor at t1. The subset of persons that admitted any self-harm both at t0 and t1 was rather small. Results further suggest that some participants showed any self-harm at t0 but not t1, and some only at t1 but not t0. Again, the findings did not differ between men and women (X^2^(3) = 3.03, *p* = 0.387, Cramer’s V = 0.12).

The groups (no self-harm at t0 & t1, self-harm at t0 & no self-harm at t1, no self-harm at t0 & self-harm at t1, self-harm at t0 & t1) did not differ in BMI before and after surgery. However, especially the group with self-harm at t0 and t1 showed a more unfavorable course of eating disorder symptoms, alcohol use, food addiction symptoms, depression symptoms, anxiety symptoms and suicidal ideation (see Table 3) as indicated by significantly higher values in this group compared to other groups after surgery but not before (except for alcohol use which was higher in this group before and after surgery). On a descriptive level, the group with self-harm at t0 and t1 (36.0%) showed a higher proportion of men than the remaining groups (self-harm at t0: 20.7%, self-harm at t1: 16.7%, self-harm at neither t0 nor t1: 24.3%).

**Table 3:**
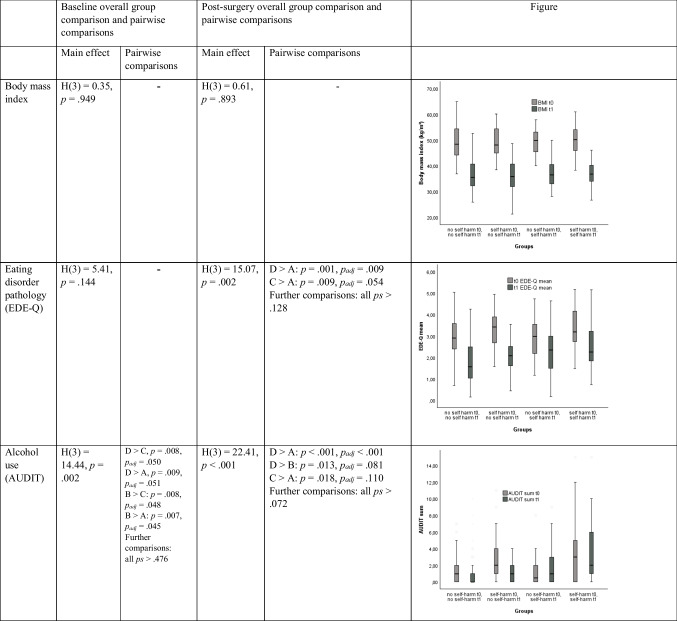

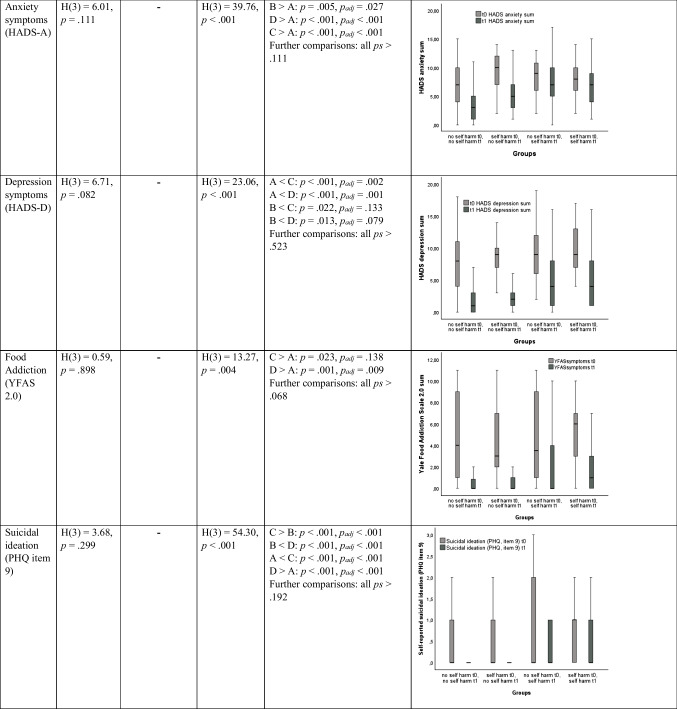
Comparisons between groups (A = no self-harm at t0 and t1, B = self-harm only at t0, C = self-harm only at t1, D = self-harm at t0 and t1) baseline and follow-up

*Notes*: Outliers are not depicted in the figures. It serves the purpose of visualization of the effects described in the table only

### Relationship of Self-Harm with BMI and Other Variables

SHI scores at t0 were significantly correlated with SHI scores at t1 (*r*_*s*_ = 0.28, *p* < 0.001) in the total sample, with a stronger effect in men (*r*_*s*_ = 0.50, *p* < 0.001) than in women (*r*_*s*_ = 0.21, *p* = 0.007; men vs. women, *z* = 2.02, *p* = 0.04). No significant correlations were found between SHI and BMI, neither at t0 nor at t1, as indicated by low correlation coefficients ranging between 0.03 and 0.06 in the total sample, -0.13 and -0.22 for men, and 0.08 and 0.15 for women.

Table 4 shows the correlations of self-harm with the other psychopathological variables for the total sample, and separately for men and women. In the total sample, most significant correlations were only small to moderate. The SHI total score at t0 was correlated with the AUDIT scores at t0 and t1 and the YFAS 2.0 at t1. The SHI total score at t1 was correlated with the AUDIT and YFAS 2.0 at t1 but not t0. As mentioned above, AUDIT means indicated a decrease in alcohol use from t0 to t1. In contrast, the SHI item 6 ‘abused alcohol’ was answered with ‘yes’ almost unchanged by eight patients before and nine patients after surgery, whereby only 3 persons answered the item this way at both time points (ĸ = 0.33 indicates fair agreement).

**Table 4:** Correlations of SHI scores with other psychopathological variables pre-surgery (t0) and six months after surgery (t1) in the total sample, and separately for men and women

	Total sample *N* = 216		Men *n* = 52		Women *n* = 164	
	SHI t_0_	SHI t_1_	SHI t_0_	SHI t_1_	SHI t_0_	SHI t_1_
Pre-surgery						
EDE-Q t_0_	.11	.03	.03	−.09	.18	.07
YFAS t_0_	.04	−.06	.00	−.20	.06	.00
AUDIT t_0_	.28^*^	.05	.33	.13	.26^*^	.03
HADS-A t_0_	.14	.05	.20	.12	.13	.03
HADS-D t_0_	.15	.14	.21	.23	.14	.11
PHQ-S t_0_	.08	.10	.09	.10	.06	.10
Post-surgery						
EDE-Q t_1_	.15	.19^*^	.17	.23	.15	.18
YFAS t_1_	.19^*^	.28^*^	.09	.23	.23^*^	.30^*^
AUDIT t_1_	.25^*^	.27^*^	.25	.38^*^	.24^*^	.25^*^
HADS-A t_1_	.20^*^	.36^*^	.22	.39^*^	.21^*^	.35^*^
HADS-D t_1_	.13	.28^*^	.20	.30	.10	.28^*^
PHQ-S t_1_	.18^*^	.51^*^	.27	.64^*^	.14	.46^*^

*N/n* based on listwise deletion; *SHI* Self-Harm Inventory; *EDE-Q* Eating Disorder Examination Questionnaire mean; *YFAS* Yale Food Addiction Scale; *AUDIT* Alcohol Use Disorder Identification Test; *HADS-A* Hospital Anxiety and Depression Scale, subscale anxiety; *HADS-D* Hospital Anxiety and Depression Scale, subscale depression; *PHQ-S* Patient Health Questionnaire-9, item 9, suicidality; *t*_*0*_ Before obesity surgery; *t*_*1*_ After obesity surgery; ^*^p_*adj.*_ < .008 (0.05/6, two-tailed)

The strong link between the number of self-harm behaviors (SHI_Total_) and both HADS-D means and the affirmative responses in the PHQ-9 suicidality item (not dichotomized) after surgery (t1), but not before surgery (t0), should be emphasized. Although it may seem so at first glance, the* z* statistics did not indicate significant differences of the t1 correlations between the SHI_Total_ and PHQ-9 suicidality item for men (*r*_*s*_ = 0.64) and women (*r*_*s*_ = 0.46; *z* = 1.60, *p* = 0.11). Likewise, t1 correlations between the SHI_Total_ and AUDIT (*z* = 0.95, *p* = 0.34) or the SHI_Total_ and YFAS 2.0 (*z* =  − 0.46, *p* = 0.64) did not differ between men and women.

## Discussion

The main outcome of the current study is that we found improvements in BMI, eating pathology, alcohol use, anxiety, depression and suicidal ideations but no changes in self-harm from before to six months after obesity surgery. While the first result is in line with expectations and fits the literature [5, 25, 28, 29, 33], the second result deserves a more detailed discussion.

It appears that the surgery was associated with neither an increase nor a decrease in self-harm. The relatively low frequency of self-harm behaviors may have contributed to the lack of differences between t0 and t1. Only one quarter of the sample admitted any self-harm both before and after surgery, which is below the SHI lifetime prevalence rates reported by [22] and [21] from their pre-surgery samples. The differences are probably due to the fact that, unlike in the previous studies, we did not ask about lifetime prevalence, but that we assessed self-harm during the past four weeks. Of particular interest are the findings displayed in Fig. 1, indicating a lack of sustained self-harm in one quarter of the participants. It appears that a group of patients who initially had reported self-harm before surgery did not report any self-harm at follow-up, and another group vice versa. The two subgroup-specific opposing changes of self-harm behaviors may have neutralized differences on sample level. Another important result is that the majority of participants showed no self-harm at either t0 or t1, which suggests that most obesity surgery patients do not tend to harm themselves. Still, the subgroup reporting self-harm behaviors at t0 and t1 benefited from obesity surgery regarding weight loss but showed worse results with regards to alcohol use, eating disorder symptoms, depression and anxiety symptoms, food addiction symptoms and suicidal ideation than other groups.

The pre-post results on suicidal ideations (item 9 of the PHQ depression module) and suicide attempts (SHI item 18) are also worth mentioning. It is not surprising that hardly any participant reported suicidal attempts pre-surgery, as acute suicidality was an exclusion criterion in this study. Sixteen percent of the sample, however, reported having suicidal ideations at baseline. Fortunately, the proportion of patients with suicidal thoughts after surgery was much lower (2.7%) and none of the participants mentioned suicide attempts after surgery, which corresponds with improved HADS-D scores at follow-up. At the same time, the relatively strong positive correlations of the SHI_Total_ with both HADS means and the affirmative responses in the PHQ suicidality item are striking. The close links can be seen as an indication of the validity of the findings regarding post-surgery suicidal ideations, anxiety/depressive symptoms and self-harm. Moreover, post-surgery self-harm was related to post-surgery addiction-like eating and alcohol use. These results once again call for consistent psychosocial surveillance of obesity surgery patients and for the inclusion of mental health professionals in the interdisciplinary obesity surgery team [55–57]. An important target group might be persons with persistent self-harm behaviors before and after obesity surgery as this group reported less or no amelioration in eating disorder symptoms, alcohol use, food addiction symptoms, depression symptoms, anxiety symptoms and suicidal ideation than persons that reported no self-harm.

### Limitations

The present study has some shortcomings. First, while the SHI addresses a broad range of self-harm behaviors [58], it does not capture frequency, intensity, onset or reasons for self-harm behaviors [59]. Second, in view of the low frequency of self-harm behaviors in our study, the sample size was probably too small to address differences between men and women. Also, the sample size was rather small for the subgrouping analysis (self-harm before surgery, after surgery, at both time points or no self-harm) potentially limiting the representativeness of the results.

The follow-up period of six months may have been too short as self-harm can occur later after surgery. It is known from large scale evidence that suicide after bariatric surgery seems to be subject to a temporal dynamic [60]: Whereas only 10% of suicides after obesity surgery took place less than one year after obesity surgery [60], 29% of suicides occurred less than two years, and 68% less than three years after obesity surgery [60]. This indicates, that the risk of suicide seems to be rather low six months after obesity surgery but seems to increase as time goes by. The assumption is further supported by a rather recent study involving five Nordic countries in which only 27% of suicides took place in the first three years after obesity surgery [61]. Applying this temporal dynamic to self-harm behaviors, one would expect self-harm behaviors not to increase on a short-term basis but to increase in the long run. Support for this assumption comes from a register-based study in which the highest number of self-harm emergencies occurred 6 quarters (i.e., 1.5 years) after obesity surgery [15]. From this point in time on, self-harm emergencies seem to tend to occur more often than in the first five quarters after obesity surgery [15]. Based on this research, it should be considered to capture follow-up periods of three or more years to capture the time period after obesity surgery in which an increase in self-harm behaviors is the most likely.

Fourth, only 220 of the 419 patients who took part in the baseline assessment at t0 [37] also answered the questionnaires at t1. We reminded the patients several times via e-mail and asked them to participate in the follow-up assessment. The drop-out rate might have been lower if the follow-up assessment had not been carried out via email but had been linked to the clinical follow-up examinations in the obesity centers.

Finally, including a non-surgery control group would have provided further insights into the course of self-harm in individuals with obesity. This group should be matched to the obesity surgery group in terms of BMI and core sociodemographic variables [21]. This would have offered another opportunity to compare results with a group that did not undergo surgery. Comparisons with control groups could be further enhanced by installing an active control group that for instance is undergoing conservative weight loss therapy (e.g., [62]). Generally, one could hypothesize that the subgroup of persons with obesity intending to undergo obesity surgery might differ from the entity of persons with obesity (e.g., with regards to psychopathological characteristics). Müller and colleagues [21] however found that lifetime self-harm behaviors and suicide attempts were not more prevalent in pre-surgical group compared to a respective population-based control group with obesity.

### Conclusions, Clinical Implications and Implications for Further Research

Taken together, our results indicate that more than half of individuals seeking surgical treatment for obesity do not report any self-harm behaviors pre-surgery. Furthermore, there does not appear to be an increase in self-harm shortly after surgery. Nevertheless, a non-negligible number of obesity surgery patients reports self-harm before and/or following surgery. Screening for self-harm, including suicidal ideations, should be part not only of preoperative psychosocial evaluations but also of postoperative care.

Before obesity surgery, binge eating is a common way to cope with aversive emotional states [63] which in most cases cannot be carried out after obesity surgery due to the anatomical restrictions. As part of exploratory analyses, global eating disorder pathology was found to be reduced after obesity surgery which is in line with research showing reduced prevalence estimates of binge eating disorder or binge eating behavior after obesity surgery [64, 65]. This implies a challenge for emotion regulation in a subset of obesity surgery patients that previously relied on binge eating to cope with aversive emotional states. It is at this point that psychotherapy is to help these patients establish a new and functional emotion regulation strategy and prevent them from using dysfunctional coping strategies such as self-harm behaviors. In obesity surgery candidates, third-wave strategies involving mindfulness-based interventions or dialectical behavior therapy interventions in group setting have been initially shown to be effective [66–69]. Group-based interventions with a rather small number of sessions might be an efficient therapy service to be applied to a variety of patients with need.

Furthermore, the study at issue highlighted the close association between pre-surgical and post-surgical self-harm behaviors. Pre-surgical self-harm behaviors were already described as a risk factor for post-surgical self-harm behaviors in another study that intended to find a risk algorithm for post-surgery psychological complications [9]. Lifetime self-harm history should be proactively explored in psychosocial evaluations prior to obesity surgery. This screening would then help to identify patients with need for consistent long-term monitoring or tailored clinical interventions.

There are several implications for future research in the field of obesity surgery. More large-scale longitudinal studies that directly assess self-harm behaviors should be carried out. Moreover, multiple dimensions of self-harm behaviors such as the type of self-harm behavior (e.g., cutting, hitting), its frequency, intensity (e.g., the depth of cuts), potential consequences (e.g., necessary surgical care), and affected body parts should be examined in future investigations. More comprehensive instruments are e.g., the Self-Injury Questionnaire [70] or the Questionnaire concerning Self-Harming Behavior that capture consequences of self-harm behaviors [71], social influences, regulation of feelings and body sensations [70]. There are also interviews that focus on self-harm behaviors such as the Suicide Attempt Self-Injury Interview [72] or the Self-Injurious Thoughts and Behaviors Interview [73]. The body part (e.g., head vs. arm) where self-harm is committed also matters with respect to evaluating different forms of self-harm behaviors and its severity. Incorporating all these aspects of self-harm behaviors and prioritizing them is important for clinical purposes as e.g., psychotherapeutic approaches would primarily focus on reduction of severe self-harm behaviors directed towards the head of patients before intending to reduce less severe self-harm behaviors directed towards the arm.

Further studies should examine potential underlying mechanisms in persons that showed persistent self-harm behavior or self-harm behavior after obesity surgery. Among potential mechanisms explaining an increase in self-harm behaviors, there are brain-gut connections. Obesity surgery alters also the way medication and alcohol is metabolized “which might increase disinhibition and impulsivity, leading to self- harm” ([74], p. 551). Another discussed mechanism might be a change in maladaptive coping strategies: Obesity surgery prevents patients from binge eating so that some patients lose their maladaptive coping strategy. Consequently, another coping strategy needs to be implemented. A case report describes the case of a young woman who showed binge eating behaviors before obesity surgery and self-harm behaviors after obesity surgery [75]. The above-mentioned case reminds of the concept of addiction transfer [76]. However, addiction transfer is a rather controversial concept with evidence speaking in favor and against this concept [77, 78]. Understanding the mechanisms of self-harm behaviors after obesity surgery is also needed for conceptualizing therapy services accordingly.

## Data Availability

Data are available from the authors upon reasonable request.
